# Regional and social inequalities in the performance of Pap test and screening mammography and their correlation with lifestyle: Brazilian national health survey, 2013

**DOI:** 10.1186/s12939-016-0430-9

**Published:** 2016-11-17

**Authors:** Mariza Miranda Theme Filha, Maria do Carmo Leal, Elaine Fernandes Viellas de Oliveira, Ana Paula Esteves-Pereira, Silvana Granado Nogueira da Gama

**Affiliations:** 1 Department of Epidemiology and Quantitative Methods in Health, Sérgio Arouca National School of Public Health, Oswaldo Cruz Foundation, Rio de Janeiro, RJ Brazil; 2 Escola Nacional de Saúde Pública Sérgio Arouca-ENSP/FIOCRUZ, Rua Leopoldo Bulhões 1480 sala 813, Manguinhos, 20041-210 RJ Brazil

**Keywords:** Mammogram, Breast cancer screening, Cervical cancer screening, Lifestyle, Health self-assessment

## Abstract

**Background:**

Mass population screening for the early detection of cervical and breast cancer has been shown to be a safe and effective strategy worldwide and has reduced the incidence and mortality rates of these diseases. The aim of this study is to analyse the reach of screening tests for cervical and breast cancer according to sociodemographic variables and to analyse their correlation with a healthy lifestyle.

**Methods:**

We have analysed data collected from 31.845 women aged 18 and over, who were interviewed for the Brazilian National Health Survey, a nationwide household inquiry, which took place between August 2013 and February 2014. The Pap tests performed in the last 3 years in women aged between 25 and 64 and screening mammogram performed in the last 2 years in women aged between 50 and 69 were considered adequate. We identified habits that constitute a healthy lifestyle, such as the consumption of five or more daily servings of fruits and vegetables, 30 min or more of leisurely physical activity and not smoking.

**Results:**

We observed that the Pap test (78.8 %) was more widespread than the screening mammogram (54.5 %), with significant geographical and social differences concerning access to health care. Access for such screening was higher for women living in more developed regions (Southeast and South), who were white-skinned, better educated, living with a partner and, especially, who were covered by private health insurance. Those who underwent the tests according to established protocols also had a healthy lifestyle, which corroborates the healthy behaviour pattern of damage prevention.

**Conclusion:**

Despite the progress made, social disparity still defines access to screening tests for cervical and breast cancer, with women covered by private health insurance tending to benefit the most. It is necessary to reduce social and regional inequalities and ensure a more uniform provision and access to the tests, especially for socially disadvantaged women, in order to reduce the incidence and mortality rate resulting from the aforementioned diseases.

## Background

The mass population screening for early detection of cervical and breast cancer, the Pap test and screening mammogram, has been shown to be a safe and effective strategy worldwide, and is considered the main reason for the reduction of mortality rates for these diseases [1, 2].

In Brazil, actions to control cervical and breast cancer started taking place in the early 1980s. In the late 1990s the National Breast and Cervical Cancer Control Program was founded [3]. The Brazilian Ministry of Health recommends that at least 70 % of women aged between 50 and 69 undergo a screening mammogram every 2 years; and 80 % of the female population aged between 25 and 64, undergo a Pap test every 3 years [4, 5].

According to Globocan 2012’s data, the International Agency for Research on Cancer (IARC), breast and cervical tumours are the most common types of cancer in Brazilian women, with incidence rates of 59.5/100,000 and 16.3/100,000, respectively [6].

Some factors that contribute to cervical cancer, such as the early initiation of sexual activity, low socioeconomic status, use of oral contraceptives and marital status are well documented in literature. With regards to breast cancer, the risk of developing the disease increases according to age, reproductive history, endocrine and genetic factors. These two types of cancer are also associated with behavioural factors. While alcohol consumption, excess weight and postmenopausal obesity increase breast cancer risk, smoking increases cervical cancer risk (directly related to the amount of cigarettes smoked) [7, 8].

Breast and cervical cancer control is a priority on the country’s health agenda and is part of the Strategic Action Plan to Tackle Chronic Non-communicable Diseases (NCDs) launched by the Ministry of Health in 2011 [9]. Efforts to increase the accessibility of preventative tests for these diseases have proven effective in reducing mortality rates of cervical cancer, but not of breast cancer, which reveals the persistence of social and regional inequalities in mortality from both diseases [10]. The aim of this study is to analyse the reach of screening tests for cervical and breast cancer and to analyse their correlation with a healthy lifestyle.

## Methods

The Brazilian National Health Survey is a nationwide household inquiry. It was carried out between August 2013 and February 2014, in a partnership with the Ministry of Health, the Oswaldo Cruz Foundation and the Brazilian Institute of Geography and Statistics (IBGE). This survey is part of the IBGE Integrated Household Surveys System and used a subsample of IBGE Master Sample, with the primary selection units (PSUs) consisting of one or more census tracts. The cluster sampling design was chosen in three selection stages (PSU, home, adult resident) with the stratification of the primary sampling units.

At all stages, the simple random sampling method was used. The most qualified person residing in the selected households provided information about sociodemographic characteristics, access and usage of health services, and private health insurance coverage about all residents in the household. In each household one resident aged 18 years or older was randomly selected for the individual interview. This interview consisted of nine modules, namely: job characteristics and social support; self-reported health status; lifestyles; chronic non-communicable diseases; traffic and labour-related accidents and violence; women's health; maternal and child care; oral health and lastly, medical care. In total, there were 60,202 interviews. Due to the cluster sampling design the results were weighted to account for this effect. Further details on sampling and data collection can be found in the 2013 National Health Survey Report [11].

In this article we have included interviews from 31,845 women aged 18 and over, representing 52.9 % of the study population. We have analysed the women's health module, focusing on the performance of preventative breast cancer (screening mammogram) and cervical cancer (Pap test) examinations, the lifestyle module and the self-reported health status module. Information about the performance of preventative examinations was reported by women themselves in order to ensure better accuracy in response. Sociodemographic information as age, skin colour (white, black, brown), years of schooling (≤7, 8–10, ≥ 11), marital status (living or not with a partner) and household location (capital and non-capital) was obtained from the household questionnaire.

In the lifestyle analysis the following habits were considered: smoking (never smoked; former smoker; smokes, but not daily; smokes every day); leisurely physical activity (engaged/not engaged in leisurely physical activity at the recommended levels−150 min or more of mild/moderate physical activity or 75 min or more of vigorous physical activity per week); recommended consumption of fruit and vegetables (consumed/did not consume at least five daily servings of vegetables and fruit).

The self-reported health status was assessed by asking the question- “In general, how do you evaluate your health?” with five options as possible responses: very good, good, fair, poor or very poor. For analysis purposes we then grouped the answers into three categories: very good or good, fair and poor or very poor.

The Pap test was considered appropriate when performed at least once in the last 3 years among women aged between 25 and 64. In relation to the screening mammogram, it was considered appropriate that women aged between 50 and 69 should have carried out the screening within the last 2 years. Both definitions followed the criteria established by the Ministry of Health [4, 5]. In the Brazilian National Health Survey, information about screening mammogram was measured on two ways: by the most qualified person residing in selected households (proxy respondent) and by the interviewee aged 18 and over (self-reported). In the current analysis, we chose to use only self-reported data, which is considered more reliable [12]. The self-reported information was preceded by the question “Have a doctor ever requested you to undergo a screening mammogram?”. Only women who had answered affirmatively were asked if it was performed and when. Thus, the probability of having had a screening mammogram was conditioned to a previous medical consultation.

Variables such as sociodemographics, self-reported health, lifestyle, and undergoing preventative screening of cervical cancer and screening mammogram were analysed according to geographical region, using the chi-square test to verify the homogeneity of proportions, considering a significance level of 5 %.

To evaluate the effect of variables such as sociodemographics and self-reported health status on the performance of preventative screening for cervical cancer and screening mammogram, a logistic regression analysis took place. The crude odds ratio was calculated and adjusted for all the variables considered in crude regression at significance level of 5 %.

To examine the association between healthy behaviour and the performance of preventative screening for cervical cancer and mammography, crude and multiple logistic regression models with the following independent variables were used: i) did not smoke (adding the options never smoked and former smoker); ii) engaged in leisurely physical activity at the recommended level; iii) consumed five or more daily servings of fruit and vegetables. We calculated the crude OR and adjusted them according to geographical region, household location, age, years of schooling, and their 95 % confidence intervals.

The Brazilian National Health Survey was approved by National Commission of Ethics in Research (CONEP) in June 2013, Regulation No. 328.159, taking into account all the recommendations of the Resolution 466/2012 of the National Health Council.

## Results

All variables showed significant regional differences—the North and Northeast had the highest proportions of people who were young, single, and brown-skinned, with lower levels of education. In the South and Southeast there was a concentration of older, better educated women who were married and white-skinned. As for private health insurance, we have identified large regional inequalities—the amount of women from the North and Northeast with private health insurance was proportionally 50 % lower than in other regions (Table 1).

**Table 1:** Characteristics of women aged 18 and over according to geographic region of residence. Brazilian National Health Survey, 2013

Variables	North (*n* = 2298)		Northeast (*n* = 8504)		Southeast (*n* = 14,049)		South (*n* = 4675)		Midwest (*n* = 2319)		Total (*n* = 31,845)		*p* value
	*N*	%	*N*	%	*N*	%	*N*	%	*N*	%	*N*	%	
Age bracket (years)													
18–29	761	33.1	2304	27.1	3123	22.2	1104	23.6	627	27.0	7918	24.9	<0.001
30–39	555	24.2	1886	22.2	2931	20.9	941	20.1	499	21.5	6812	21.4	
40–49	400	17.4	1557	18.3	2592	18.4	911	19.5	441	19.0	5902	18.5	
50–59	274	11.9	1225	14.4	2429	17.3	788	16.8	369	15.9	5085	16.0	
≥60	308	13.4	1533	18.0	2974	21.2	930	19.9	382	16.5	6127	19.2	
Marital Status													
Living with a partner	1425	62.0	4963	58.4	7900	56.2	2992	64.0	1385	59.7	18,666	58.6	<0.001
Not living with a partner	873	38.0	3541	41.6	6148	43.8	1683	36.0	933	40.3	13,179	41.4	
Skin colour													
White	511	22.7	2306	27.6	7943	57.4	3618	78.0	923	40.4	15,301	48.8	<0.001
Black	175	7.8	1015	12.1	1357	9.8	219	4.7	167	7.3	2933	9.4	
Brown	1562	69.5	5039	60.3	4540	32.8	800	17.3	1139	52.2	13,133	41.9	
Years of Schooling													
≤7	907	39.5	3886	45.7	4748	33.8	1804	38.6	796	34.3	12,141	38.1	<0.001
8–10	383	16.7	1183	13.9	2000	14.2	731	15.6	366	15.8	4662	14.6	
≥11	1008	43.9	3436	40.4	7300	52.0	2140	45.8	1157	49.9	15,041	47.2	
Women covered by private health insurance													
Yes	388	16.9	1502	17.7	5619	40.0	1602	34.3	772	33.3	9882	31.0	<0.001
No	1911	83.1	7002	82.3	8430	60.0	3073	65.7	1546	66.7	21,963	69.0	
Self-reported health status													
Very good or good	1322	57.5	4455	52.4	9528	67.8	3078	65.8	1503	64.8	19,886	62.4	<0.001
Fair	800	34.8	3248	38.2	3792	27.0	1295	27.7	677	29.2	9812	30.8	
Poor or very poor	176	7.6	801	9.4	729	5.2	302	6.5	139	6.0	2146	6.7	
Smoking													
Never smoked	1809	78.7	6325	74.4	10,555	75.1	3396	72.6	1769	76.3	23,855	74.9	<0.001
Former smoker	309	13.5	1337	15.7	1876	13.4	657	14.1	308	13.3	4488	14.1	
Smokes, but not daily	47	2.0	140	1.7	162	1.2	57	1.2	23	1.0	429	1.3	
Smokes every day	133	5.8	702	8.3	1456	10.4	565	12.1	218	9.4	3074	9.7	
Leisurely physical activity at the recommended levels^a^													
No	1943	84.6	7005	82.4	11,370	80.9	3832	82.0	1850	79.8	26,000	81.6	0.047
Yes	355	15.4	1499	17.6	2678	19.1	844	18.0	469	20.2	5845	18.4	
Recommended consumption of fruit and vegetables^b^													
No	1476	64.2	6115	71.9	7928	56.4	2945	63.0	1223	52.7	19,687	61.8	<0.001
Yes	822	35.8	2389	28.1	6121	43.6	1730	37.0	1096	47.3	12,158	38.2	
Mammogram (50–69 years old)													
Never	257	54.8	929	45.2	953	23.3	348	26.6	210	34.3	2697	31.6	<0.001
Last than two years ago	155	33.1	860	41.9	2550	62.3	785	59.9	298	48.6	4647	54.5	
Two years ago or more	57	12.2	265	12.9	590	14.4	176	13.5	104	17.0	1192	14.0	
Pap test (25–64 years old)													
Never	213	12.8	739	12.3	899	8.9	222	6.6	133	7.8	2205	9.7	<0.001
Last than 3 years ago	1243	75.1	4480	74.4	8120	80.5	2780	82.7	1372	80.7	17,994	78.8	
Three years ago or more	201	12.1	805	13.4	1069	10.6	360	10.7	194	11.4	2629	11.5	

^a^at least 150 min or more of mild/moderate physical activity or 75 min or more of vigorous physical activity per week

^b^at least five daily servings of vegetables and fruit

Healthy habits also varied according to the geographical region. The proportion of women who smoked daily was 12.1 % in the South, which is much higher than the national average (9.7 %), while the Northern region had the lowest proportion of women who smoked (5.8 %). The engagement in physical activity during leisure time, although proven to be low in Brazil (18.4 %), also showed regional differences, being more frequent in the Midwest and Southeast, and with lesser participation in the North. Similarly, the daily consumption of fruit and vegetables was lower in the Northeast, in contrast to the Midwest and Southeast, regions in which almost 50 % of women consumed at least five servings of these foods a day. Most women rated their own health as “very good” or “good” and 6.7 % considered it “poor” or “very poor”, but regional differences remained, with the worst self-reported health in the North and Northeast regions (Table 1).

In women between 50 and 69, almost a third had never undergone a screening mammogram, whilst in the North this proportion exceeded 50 %. The participation in at least one preventative examination for cervical cancer was more frequent, reaching over 90 % among Brazilian women aged between 25 and 64, with lower proportions for the North and Northeast regions. Considering the timely implementation of mammography, 54.5 % had this exam done less than 2 years ago. In the North, however, only 33.1 % had been screened. As for preventative screening for cervical cancer, 78.8 % reported having been screened less than 3 years ago, without any significant regional differences (Table 1).

Table 2 shows the relationship between sociodemographic variables and the performance of preventative screening for cervical cancer and screening mammogram, according to the protocol established by the Ministry of Health. Older women underwent fewer preventative examinations than younger ones did, most often in the age group of 35–44. Married women, women with higher levels of education and with private health insurance also were more likely to have a Pap test as recommended. For those covered by private health insurance, accessibility rose by more than 200 % (adjusted OR = 2.49). Considering the crude analysis, the geographical area of residence, the location of the woman's home and skin colour showed a link with the completion of preventative screening for cervical cancer, however, they lost statistical significance after adjusting for the other variables.

**Table 2:** Crude and adjusted Odds Ratio for Pap test and screening mammogram. Brazilian National Health Survey, 2013

Variables	Pap tests performed in the last 3 years in women aged between 25 and 64				Screening mammogram performed in the last 2 years in women aged between 50 and 69			
	OR crude (95 % CI)	*p* value	Adjusted OR^a^ (95 % CI)	*p* value	OR crude (95 % CI)	*p* value	Adjusted OR^b^ (95 % CI)	*p* value
Region		<0.001		0.066		<0.001		<0.001
North	0.73 (0.61–0.87)		0.93 (0.77–1.11)		0.30 (0.23–0.38)		0.35 (0.27–0.46)	
Northeast	0.70 (0.61–0.81)		0.93 (0.81–1.08)		0.44 (0.37–0.52)		0.56 (0.46–0.69)	
Southeast	1		1		1		1	
South	1.16 (0.96–1.40)		1.16 (0.96–1.41)		0.91 (0.72–1.13)		1.05 (0.83–1.41)	
Midwest	1.02 (0.87–1.19)		1.13 (0.94–1.29)		0.57 (0.47–0.71)		0.59 (0.47–0.74)	
Household location		<0.001		0.29		<0.001		0.014
Capital	1.43 (1.25–1.65)		1.08 (0.93–1.26)		2.22 (1.82–2.71)		1.34 (1.06–1.70)	
Non-capital	1		1		1		1	
Age bracket (years)		<0.001		<0.001				
25–34	1.52 (1.30–1.77)		1.28 (1.08–1.51)		–		–	
35–44	1.97 (1.70–2.28)		1.70 (1.45–1.99)		–		–	
45–54	1.78 (1.52–2.09)		1.70 (1.43–2.01)		–		–	
55–64	1		1		–		–	
Years of Schooling		<0.001		<0.001		<0.001		<0.001
≤7	1		1		1		1	
8–10	1.35 (1.15–1.59)		1.19 (1.00–1.41)		2.03 (1.59–2.58)		1.70 (1.28–2.23)	
≥11	2.20 (1.95–2.49)		1.56 (1.35–1.80)		3.02 (2.53–3.60)		1.99 (1.62–2.44)	
Marital Status		<0.001		<0.001		<0.001		<0.001
Living with a partner	1.86 (1.66–2.08)		1.85 (1.64–2.01)		1.44 (1.24–1.67)		1.54 (1.31–1.80)	
Not living with a partner	1		1		1		1	
Skin colour		<0.001		0.037		<0.001		0.772
White	1		1		1		1	
Black	0.74 (0.60–0.91)		1.01 (0.81–1.25)		0.66 (0.51–0.85)		1.07 (0.80–1.41)	
Brown	0.66 (0.59–0.75)		0.86 (0.75–0.97)		0.63 (0.538–0.732)		1.07 (0.89–1.28)	
Women covered by private health insurance		<0.001		<0.001		<0.001		<0.001
Yes	3.21 (2.76–3.72)		2.51 (2.13–2.96)		3.75 (3.11–4.51)		2.54 (2.07–3.11)	
No	1		1		1		1	
Self-reported health status		<0.001		0.234		<0.001		0.090
Very good or good	1.80 (1.49–2.17)		1.19 (0.97–1.45)		1.80 (1.43–2.28)		1.01 (0.77–1.32)	
Fair	1.29 (1.06–1.56)		1.15 (0.94–1.40)		1.07 (0.85–1.36)		0.84 (0.64–1.09)	
Poor or very poor	1		1		1		1	

^a^Adjusted by region, household location, age, schooling, marital status, skin colour, coverage by private health insurance and self-reported health status

^b^Adjusted by region, household location, schooling, marital status, skin colour, coverage by private health insurance and self-reported health status

The performance of screening mammogram has statistically shown a significant link with most of the sociodemographic variables (Table 2). Women who lived with a partner and women who lived in urban areas were more likely to undergo a screening mammogram than women in a different situation. Having been in education for 11 years or more, and possessing private health insurance, doubled the chances of women being assessed for breast cancer according to protocol. On the other hand, residing in the North, Northeast and Midwest reduced the chance of having the examination performed properly. In the North, this reduction was of 65 % (adjusted OR = 0.35).

The results of logistic regression have shown an association between lifestyle choices and whether the preventative screening for cervical cancer or mammography was carried out properly (Tables 3 and 4). Women who did not smoke, who engaged in regular leisurely physical activity and who consumed the recommended amounts of fruit and vegetables were more likely to routinely undergo preventative cervical cancer screening (Table 3). The same pattern was observed for those who underwent mammography according to protocol (Table 4). All three lifestyle choices remained statistically significant associated with both screenings after controlling for the confounders region of residence, age, years of schooling and household location. (Tables 3 and 4).

**Table 3:** Healthy lifestyle associated with Pap test screening performed in the last 3 years in women aged between 25 and 64. Brazilian National Health Survey, 2013

Variables	Pap test screening performed in the last 3 years in women aged between 25 and 64					
	Crude OR (IC 95 %)	*p*-value	Adjusted OR^a^ (IC 95 %)	*p*-value	Adjusted OR^b^ (IC 95 %)	*p*-value
Not smoking	1.78 (1.54–20.6)	<0.001	1.83 (1.58–2.12)	<0.001	1.66 (1.43–1.92)	<0.001
Recommended leisurely physical activity^c^	2.21 (1.85–2.64)	<0.001	2.20 (1.84–2.64)	<0.001	1.96 (1.63–2.35)	<0.001
Recommended consumption of fruit and vegetables^d^	1.39 (1.24–1.55)	<0.001	1.34 (1.19–1.50)	<0.001	1.27 (1.14–1.43)	<0.001

^a^Adjusted only for region

^b^Adjusted for age, schooling, region and household location

^c^at least 150 min or more of mild/moderate physical activity or 75 min or more of vigorous physical activity per week

^d^at least five daily servings of vegetables and fruit

**Table 4:** Healthy lifestyle associated with screening mammogram performed in the last 2 years in women aged between 50 and 69. Brazilian National Health Survey, 2013

Variables	Screening mammogram performed in the last 2 years in women aged between 50 and 69					
	Crude OR (IC 95 %)	*p*-value	Adjusted OR^a^ (IC 95 %)	*p*-value	Adjusted OR^b^ (IC 95 %)	*p*-value
Not smoking	1.41 (1.15–1.74)	0.001	1.49 (1.21–1.83)	<0.001	1.59 (1.26–2.01)	<0.001
Recommended leisurely physical activity^c^	3.22 2.63–3.96)	<0.001	3.31 (2.69–4.06)	<0.001	2.85 (2.23–3.63)	<0.001
Recommended consumption of fruit and vegetables^d^	1.62 (1.40–1.86)	<0.001	1.54 (1.33–1.78)	<0.001	1.45 (1.23–1.72)	<0.001

^a^Adjusted only for region

^b^Adjusted for age, schooling, region and household location

^c^at least 150 min or more of mild/moderate physical activity or 75 min or more of vigorous physical activity per week

^d^at least five daily servings of vegetables and fruit

## Discussion

The Pap test coverage among women aged 25–64 years was almost 80 %, reaching the standard recommended by the Ministry of Health for the Brazilian population. However, for mammograms, the proportion was 54.5 %, failing to comply with the 70 % target.

There were geographic and socioeconomic differences which influenced access to the Pap test and screening mammogram. Women living in the Southeast and South, who were white-skinned, better educated, living with a partner and, especially, those covered by private health insurance had a better chance of getting screened. Those who underwent examinations according to established protocols also had a healthier lifestyle, which corroborates the healthy behaviour pattern of damage prevention—regardless of the geographical region of residence and the socioeconomic conditions.

Differences in the coverage of the Pap test by geographical regions were lower than those observed for screening mammogram. The Pap test is available as a basic ambulatory care service of the Unified Health System, being accessible to all women at no cost. In the last 20 years Brazil has greatly extended the reach of primary care, with the use of the Family Health Strategy [13]. The Brazilian National Household Survey carried out in 2008 showed that among people seeking care for health-related issues, 96.3 % were seen by a health provider in their first attempt, without major differences between the lowest and highest-income class [14], highlighting the universality of access to health services. On the other hand, the screening mammography is not available in the facilities that offer primary care. Appointments have to be scheduled at centres of specialized diagnostic support services, venues largely in demand by the private sector. Therefore, the demands of the Unified Health System are not prioritised [14, 15].

Between 2002 and 2009, Brazil doubled its number of mammography units, reaching a ratio of 48 mammography units per million women, similar to the rate found in developed countries [16]. Yet, this increase could not be matched across the country meaning the regional and social inequalities were not improved [17]. Moreover, the integration between the primary and secondary levels of care is poor, without proper ordering of assistance flows and regulation centres for consultations and examinations [18].

Therefore, access to screening mammogram for Unified Health System users, which represent 75 % of the population [19], is limited. This may explain the differences in access identified between the two types of tests in this article.

Women who were older, less educated, unmarried, brown-skinned and not covered by private health insurance were less likely to undergo a Pap test or screening mammography at the recommended intervals. This further reveals inequality in access to such tests.

In a review article of Schueler et al. (2008) [20], the characteristics associated with lower coverage of screening mammogram in 1988–2007 were similar to what we have found in this study. In Brazil, the State of São Paulo Multicenter Health Study, conducted in 2001–2002, also showed significant socioeconomic and racial inequalities in access to mammography examination [21]. The strong association between being covered by private health insurance and the proper execution of a mammography exam or a Pap test was also found in several international studies [2, 22, 23].

Despite the social and regional disparities in accessibility of screening mammogram in Brazil, the country witnessed an expansion in coverage in the last 10 years, increasing from 47.2 % in 2003 to 54.5 % in 2013. For the Pap test, the increase was even greater—from 65.5 to 78.8 % [24]. The results indicate the progress achieved by the actions taken and investments made in the country.

We can detect the effectiveness of the prevention of cervical cancer by analysing the trend of mortality from the disease. There was a steady decline in mortality rates in all regions of Brazil from 1980 to 2010 [10]. The universal provision of primary care has an important role, since early treatment of precursor injuries of this type of cancer can occur at the outpatient level.

As for breast cancer, the reduction observed in mortality is restricted to the capitals of the Southeast and South [10], which hold the largest population proportions with private health insurance, 56.3 and 54.1 % respectively [19], higher rates of screening mammogram and specialized hospital services [17, 25].

In recent years, studies have shown that the chance of screening for these two diseases is higher among women with a healthy lifestyle [26, 27], which could also explain lower incidence rates and mortality in this patient subset.

The results of this study clearly support the correlation between a healthy lifestyle and periodic health examinations. Women who engaged in physical activity during leisure time, who did not smoke and who had a healthier diet were more likely to carry out a Pap test and screening mammogram following the protocol recommended by the Ministry of Health. We observed inequality in the willingness and capability of those living in poorer regions, namely in the North and Northeast where Human Development Index is lowest, to adopt a healthier lifestyle. Nonetheless, this trend did not apply for smoking, where we observed lower levels among the poorer population.

Similar results were observed in the study carried out across a representative sample of the state of Minas Gerais, the third largest economy in Brazil, in 2003. The author found a positive relationship between having private health insurance and not smoking, engaging in physical activity and eating five or more servings of fruit and vegetables a day. Similarly, we have established a link between seeking preventative cervical and mammography screening tests within the recommended period with being covered by private health insurance [28].

The Brazilian data provided by the World Health Survey showed that there was a decrease in the proportion of women who smoke, from 14.9 % in 2003 to 9.7 % in 2013, an increase in the level of education (11 years or more of education) from 30.7 to 47.2 %, and access to private health insurance, from 25.9 to 31.0 %. All these factors contributed to improving access to preventative screening for cervical cancer and screening mammogram, as well as health self-assessment – good or very good—which increased from 48.6 to 62.4 % [24].

## Conclusion

To increase the reach and reduce inequality of access to screening tests for cervical and breast cancer, we suggest a more even distribution of health services and provision of access to these tests—particularly for women who are socially disadvantaged—in order to reduce the incidence and mortality from these diseases.

Regarding the study's weaknesses, the sources of information on screening tests were self-reported, therefore subject to memory and information bias. However, studies related to the screening of cervical and breast cancer in the United States have already shown that there is a high correlation between self-reported data and those recorded in medical records [29, 30]. Particularly in relation to screening mammogram in Brazil, self-reported data tend show a slightly lower coverage in comparison to when it is provided by the proxy respondent (60 %) [31].

As for the study’s strengths, the data is primary, population-based, representative of the country and macro-geographical.
